# Supplementary material for: Human isotype‐dependent inhibitory antibody responses against Mycobacterium tuberculosis
Source: EMBO Mol Med. 2016 Oct 11;8(11):1325–39. doi: 10.15252/emmm.201606330 (PMC5090662; doi:10.15252/emmm.201606330)
Supplement: Supplementary file 1 — Appendix [file EMMM-8-1325-s001.pdf]

Appendix

Content:

Appendix Table S1: TB patient information

Appendix Table S2: Ig gene features and antibody reactivity of plasmablast antibodies

Appendix Table S3: Ig gene features and antibody reactivity of antibodies generated from HBHA-reactive memory B cells

Appendix Table S4: Functional characterization of plasmablast antibodies

Appendix Table S5: Statistical analysis

Appendix Figure S1: Antigen-reactivity and concentration-dependent effect of monoclonal IgG and IgA antibodies on MTB infection of human lung epithelial cells.

Appendix Table S1: TB patient information

Patients			Sample	Serum Ig			Plasmablasts
				IgG	IgA	IgM	
TB	Sex	Age	Timepoint	[AUC x10^-2]	[AUC x10^-3]	[AUC x10^-3]	[%]
**7***	**F**	**32**	**BT**	**1,3**	0,6	**1,2**	**1,2**
14	M	52	AT (2 m)	**1,7**	0,5	**1,1**	0,5
15	F	33	AT (2 m)	0,3	ND	ND	**0,8**
16	M	50	BT	**1,0**	**1,0**	**1,1**	0,6
17	M	41	BT	**1,3**	**1,4**	**1,0**	0,3
18	M	68	BT	0,2	0,6	**1,1**	0,0
19	F	44	BT	**1,5**	ND	ND	0,2
20	F	58	BT	0,5	0,6	0,8	0,2
21	M	55	BT	0,7	ND	ND	**3,0**
22	M	32	AT (3 m)	**1,3**	ND	ND	**2,5**
23	M	52	BT	**1,4**	0,3	0,6	**3,1**
**24***	**F**	**61**	**BT**	**1,1**	0,6	0,7	**5,0**
25	F	38	BT	0,5	**1,0**	**1,1**	**4,7**
26	F	65	BT	**1,2**	**0,7**	**1,1**	0,1
29	M	58	AT (2 wk)	**1,1**	0,5	0,7	0,6
30	F	30	BT	**1,1**	**0,9**	0,9	0,1
31	M	50	AT (2 wk)	**1,1**	**0,8**	0,9	**1,8**
**33***	**M**	**25**	**BT**	**1,1**	0,5	0,6	**6,5**
34	F	27	AT (2 m)	0,6	ND	ND	0,1
35	?	?	BT	**1,2**	ND	ND	**1,0**
36	M	31	BT	0,6	ND	ND	**2,4**
37	M	34	AT (1 m)	0,7	ND	ND	0,0
39	M	15	BT	0,5	**0,8**	**1,1**	0,0
40	M	17	BT	0,4	0,6	**1,0**	0,6
41	M	30	AT (wk)	0,6	ND	ND	ND

TB - Tuberculosis; * - patient selected for mAb cloning; numbers in bold indicate + reactivity/PB population

BT - before treatment; AT - after treatment; m - months; w - weeks; Serum Ig - Ig serum reactivity to MTB whole cell lysate

Appendix Table S2: Ig gene features and antibody reactivity of plasmablast antibodies.

TB patient	Nr	Isotype Heavy Chain	VH	JH	SHM	CDR3	Isotype Light Chain	VL	JL	SHM	MTB lysate	MTB bacteria	Cross-reactive	MTB Cell Membrane	ManLAM	BCG bacteria (FACS)	Esat-6	Ag85B
Pat TB 7	014	IgG3	IGHV3-74*03	IGHJ3*02	7	DPPADDAFDI	lambda	IGLV3-21*02	IGLJ2*01	11	-	-	-				-	-
Pat TB 7	017	IgG1	IGHV1-69*01	IGHJ4*02	20	TAFGVVGLDFDY	lambda	IGLV7-46*01	IGLJ2*01	9	+	+	-				+	-
Pat TB 7	023	IgG1	IGHV1-8*01	IGHJ4*02	20	VEWYRPFDF	kappa	IGKV3-20*01	IGKJ4*01	9	+	-	+	+	-	-	-	-
Pat TB 7	028	IgG2	IGHV4-59*01	IGHJ6*04	15	NQWGALDV	lambda	IGLV2-8*01	IGLJ2*01	10	-	-	-				-	-
Pat TB 7	031	IgG1	IGHV3-15*07	IGHJ4*02	17	GFQGRGGWFRHITQY	kappa	IGKV1D-33*01	IGKJ3*01	8	+	+	+			+	+	+
Pat TB 7	034	IgG1	IGHV4-59*08	IGHJ4*02	24	RYGGYYFDH	kappa	IGKV1D-12*01	IGKJ2*01	9	+	-	-	+		-	-	-
Pat TB 7	038	IgG1	IGHV3-11*03	IGHJ1*01	10	GNRCTGDCFRHAEYFQH	kappa	IGKV2-28*01	IGKJ4*01	9	-	-	-				-	-
Pat TB 7	066	IgA2	IGHV3-15*07	IGHJ6*03	2	DNMDV	lambda	IGLV4-69*01	IGLJ3*02	8	+	-	-	-		-	-	-
Pat TB 7	067	IgG1	IGHV3-21*01	IGHJ6*03	1	DNLPNRYMDV	lambda	IGLV1-51*02	IGLJ3*02	11	+	-	-	+	-	+	-	-
Pat TB 7	069	IgA1	IGHV3-74*03	IGHJ4*02	24	DLSGAADY	lambda	IGLV1-47*01	IGLJ1*01	11	-	-	-				-	-
Pat TB 7	070	IgG3	IGHV-4-34*01	IGHJ6*03	19	GSVTTDSYYYYIDV	kappa	IGKV1-6*01	IGKJ1*01	9	+	-	-	+		-	-	-
Pat TB 7	072	IgA1	IGHV1-18*01	IGHJ3*02	15	DPRYSSLWYRAGDDFDT	kappa	IGKV3-20*01	IGKJ2*01	11	-	-	-				-	-
Pat TB 7	079	IgG1	IGHV3-30*01	IGHJ5*02	13	PHDIVITNPFDL	kappa	IGKV1D-16*01	IGKJ4*01	9	+	+	-	-	-	-	-	-
Pat TB 7	085	IgG1	IGHV4-b*01	IGHJ6*03	7	GYDFWSVIKYYYMDV	lambda	IGLV1-47*01	IGLJ1*01	11	-	+	-			-	+	+
Pat TB 7	101	IgG3	IGHV4-34*01	IGHJ4*02	1	RSRFSYGRQGFDY	kappa	IGKV3-20*01	IGKJ4*01	9	-	+	+				+	+
Pat TB 7	110	IgA1	IGHV1-18*01	IGHJ3*02	11	PLQHLSRSDEAFDI	lambda	IGLV3-21*02	IGLJ2*01	11	+	-	-	-		-	+	-
Pat TB 7	119	IgG2	IGHV4-59*08	IGHJ5*02	13	SIGYCSGGNCFSSGKGYNFFDP	kappa	IGKV3-20*01	IGKJ4*01	10	-	-	-	-			-	-
Pat TB 7	131	IgA1	IGHV3-66*02	IGHJ2*01	36	TTLNWYFDV	lambda	IGLV2-14*01	IGLJ1*01	12	-	+	-			-	+	-
Pat TB 7	141	IgA2	IGHV3-9*01	IGHJ4*02	18	GGRRGSWSPAFDS	lambda	IGLV2-14*01	IGLJ1*01	9	-	-	-	-		-	+	-
Pat TB 7	147	IgG1	IGHV3-23*04	IGHJ4*02	11	APFAWELLDFDY	lambda	IGLV2-14*01	IGLJ3*02	10	-	-	-	-			-	-
Pat TB 7	149	IgG1	IGHV4-4*02	IGHJ4*02	25	GSGRNYFDSIGFLSYFDF	kappa	IGKV1-9*01	IGKJ2*04	9	-	+	-	-			-	-
Pat TB 7	153	IgG3	IGHV3-66*02	IGHJ6*02	56	SSLCGGDCYSPVYFHYLDI	lambda	IGLV2-23*02	IGLJ1*01	11	+	+	+			+	+	+
Pat TB 7	159	IgA2	IGHV3-66*02	IGHJ4*02	25	DVHSSGAY	kappa	IGKV4-1*01	IGKJ1*01	9	-	-	-				-	-
Pat TB 7	168	IgA2	IGHV4-39*01	IGHJ4*02	24	HGHDNGDYHGHFFQY	kappa	IGKV4-1*01	IGKJ2*01	10	-	-	-				-	-
Pat TB 7	169	IgA2	IGHV3-74*03	IGHJ4*02	15	DLSGGRDF	kappa	IGKV2-28*01	IGKJ2*01	9	-	-	-	-			-	-
Pat TB 7	181	IgG1	IGHV4-59*01	IGHJ5*02	33	QFTPDFTSWFLEP	kappa	IGKV4-1*01	IGKJ1*01	9	-	-	-				-	-
Pat TB 7	186	IgG2	IGHV4-4*02	IGHJ4*02	12	VEAQSNSSNWPHDH	lambda	IGLV1-40*01	IGLJ3*02	11	-	-	-				-	-
Pat TB 7	194	IgG1	IGHV3-33*01	IGHJ4*02	21	GLLSFATTEIDY	lambda	IGLV1-47*01	IGLJ1*01	11	-	-	-	-			-	-
Pat TB 7	195	IgG3	IGHV4-31*03	IGHJ3*02	19	GGYDGYDAFSI	kappa	IGKV1-5*01	IGKJ2*01	9	-	-	-				-	-
Pat TB 7	196	IgG1	IGHV4-34*01	IGHJ4*02	5	DVFPLRLYYYQSSGFYNY	kappa	IGKV3-20*01	IGKJ2*02	9	+	+	+		-	+	-	+
Pat TB 7	208	IgG1	IGHV5-51*03	IGHJ3*02	19	RKLEADAIDF	kappa	IGKV1-9*01	IGKJ4*01	10	-	-	-	+	-	-	-	-
Pat TB 7	231	IgG1	IGHV3-21*01	IGHJ4*02	9	DSPLPYILTGFDY	kappa	IGKV1-17*01	IGKJ1*01	9	-	+	-				-	-
Pat TB 7	232	IgA2	IGHV3-7*01	IGHJ4*02	24	ENRGFEF	lambda	IGLV1-44*01	IGLJ3*02	11	+	-	+		+	+	-	-
Pat TB 7	233	IgG3	IGHV4-34*01	IGHJ5*02	3	DRIVVTATRWFDP	kappa	IGKV3-15*01	IGKJ1*01	8	-	+	-	-		-	+	-
Pat TB 7	242	IgG1	IGHV3-74*01	IGHJ4*02	8	SVGSGAWRHYFDY	lambda	IGLV1-40*01	IGLJ3*02	12	+	-	-	+		-	-	-
Pat TB 7	243	IgG1	IGHV3-30*13	IGHJ3*02	23	EKLAYYDADGTYYGPGALDI	kappa	IGKV3-15*01	IGKJ1*01	9	-	-	-				-	-
Pat TB 7	266	IgG1	IGHV1-3*01	IGHJ5*02	6	GGRYYGSGSLNWFDP	kappa	IGKV4-1*01	IGKJ1*01	10	-	-	+				-	-
Pat TB 7	286	IgG1	IGHV3-49*04	IGHJ4*02	14	VGSYRYFDY	kappa	IGKV3-15*01	IGKJ4*01	9	-	-	-				-	-
Pat TB 7	292	IgG3	IGHV1-2*02	IGHJ5*02	0	DRGEAPPSLVGATGNWFDP	kappa	IGKV4-1*01	IGKJ2*01	9	-	-	-				-	-
Pat TB 7	317	IgG2	IGHV3-33*03	IGHJ4*02	12	DYGTTGDLAVAGHLDY	lambda	IGLV1-40*01	IGLJ3*02	11	+	-	-	+	-	+	-	-
Pat TB 7	349	IgG1	IGHV4-59*08	IGHJ4*02	21	LFYYDGSSYDPGIDY	lambda	IGLV7-43*01	IGLJ3*02	9	+	-	-	-	-	-	-	-
Pat TB 7	354	IgG1	IGHV3-30*04	IGHJ5*02	1	DACVVKELCPPPSFDP	kappa	IGKV3-15*01	IGKJ4*01	9	-	-	-				-	-

TB patient	Nr	Isotype Heavy Chain	VH	JH	SHM	CDR3	Isotype Light Chain	VL	JL	SHM	MTB lysate	MTB bacteria	Cross-reactive	MTB Cell Membrane	ManLAM	BCG bacteria (FACS)	Esat-6	Ag85B
Pat TB33	003	IgA1	IGHV3-23*01	IGHJ4*02	6	GRYTYGYYFDY	kappa	IGKV3-20*01	IGKJ3*01	1	-	-	-					
Pat TB33	010	IgA2	IGHV1-2*02	IGHJ4*02	26	LPYITTSRDF	kappa	IGKV3-20*01	IGKJ4*01	20	+	-	+			-		
Pat TB33	026	IgA1	IGHV3-7*01	IGHJ4*02	26	DPGWGAFDY	kappa	IGKV2-30*02	IGKJ2*01	6	-	-	-					
Pat TB33	032	IgA1	IGHV3-53*01	IGHJ2*01	27	LPLGDWYFDL	lambda	IGLV2-23*02	IGLJ3*02	21	-	-	-					
Pat TB33	033	IgA1	IGHV3-74*03	IGHJ3*01	19	SKLLADDAFDV	kappa	IGKV3-20*01	IGKJ1*01	12	-	-	-					
Pat TB33	052	IgG1	IGHV3-21*01	IGHJ3*01	30	HILGVPGAFHV	kappa	IGKV1-NL1*01	IGKJ2*01	15	+	+	-	-		-		
Pat TB33	055	IgG2	IGHV3-30-3*01	IGHJ4*02	30	RGPFTTGRYDFYGEEYYFDS	kappa	IGKV1-5*03	IGKJ1*01	14	-	-	-					
Pat TB33	057	IgG1	IGHV3-9*01	IGHJ4*02	32	VSYSSGWYLDH	kappa	IGKV1-27*01	IGKJ2*01	18	-	-	-					
Pat TB33	059	IgA2	IGHV4-39*07	IGHJ4*02	16	YAKYSSTSYGAFDY	lambda	IGLV2-11*01	IGLJ3*02	5	+	-	-			-		
Pat TB33	064	IgG1	IGHV3-21*01	IGHJ4*02	15	DGRDGYNLNY	lambda	IGLV6-57*01	IGLJ3*02	6	-	-	-					
Pat TB33	066	IgA1	IGHV3-7*01	IGHJ5*02	20	DRTLAA	kappa	IGKV3D-15*01	IGKJ2*01	8	-	-	-					
Pat TB33	091	IgA2	IGHV3-21*01	IGHJ4*02	13	DPPHDYGNNAEDY	kappa	IGKV1-39*01	IGKJ1*01	12	-	-	+					
Pat TB33	093	IgA2	IGHV5-51*01	IGHJ3*01	30	IGAGDSSADAFDV	lambda	IGLV1-47*01	IGLJ1*01	28	+	-	-	-		-		
Pat TB33	103	IgG1	IGHV3-74*01	IGHJ4*02	26	ESYRRFDD	lambda	IGLV2-8*01	IGLJ1*01	3	-	-	-					
Pat TB33	114	IgA1	IGHV3-11*01	IGHJ6*02	13	EPGMERMDV	kappa	IGKV1-16*02	IGKJ2*01	9	-	-	-					
Pat TB33	115	IgA1	IGHV1-3*01	IGHJ3*02	26	TRPYDVLSSLDDAFDL	kappa	IGKV1-39*01	IGKJ4*01	23	-	-	-					
Pat TB33	117	IgA2	IGHV3-15*01	IGHJ4*02	21	SAAAGLPDC	lambda	IGLV1-51*01	IGLJ3*02	8	+	-	+					
Pat TB33	123	IgA1	IGHV3-48*02	IGHJ5*02	29	VGNSAAPDW	lambda	IGLV3-27*01	IGLJ3*02	9	-	-	-		+			
Pat TB33	126	IgG2	IGHV3-21*01	IGHJ4*02	24	DILGASRGFDY	kappa	IGKV3-11*01	IGKJ4*01	8	-	-	-					
Pat TB33	127	IgA1	IGHV7-4-1*02	IGHJ5*02	36	WSCSSGNCYLGH	kappa	IGKV3-20*01	IGKJ4*01	13	-	-	-					
Pat TB33	129	IgA2	IGHV4-39*07	IGHJ6*02	9	RGSGFNGMDV	kappa	IGKV3-20*01	IGKJ2*01	3	+	-	-	-		-		
Pat TB33	137	IgA2	IGHV4-59*01	IGHJ4*02	10	EPSWSGYFDY	kappa	IGKV3-20*01	IGKJ1*01	7	-	-	-					
Pat TB33	138	IgA2	IGHV3-30*18	IGHJ6*02	18	DFIVLDYDEHYGMDV	lambda	IGLV1-44*01	IGLJ3*02	25	-	+	-					
Pat TB33	141	IgG1	IGHV4-31*03	IGHJ4*02	17	GDWNDVHVDF	lambda	IGLV1-40*01	IGLJ1*01	9	-	-	-					
Pat TB33	146	IgG2	IGHV4-39*01	IGHJ5*02	25	GLYNSDWLDP	lambda	IGLV1-51*01	IGLJ3*02	15	-	-	-					
Pat TB33	148	IgA2	IGHV3-73*02	IGHJ4*02	22	RIPNVNDF	lambda	IGLV2-14*01	IGLJ3*02	13	+	+	+					
Pat TB33	152	IgG2	IGHV1-2*02	IGHJ5*02	9	ELGSTGGHWFDP	kappa	IGKV3-20*01	IGKJ1*01	6	-	-	-					
Pat TB33	161	IgA2	IGHV4-39*02	IGHJ4*02	36	DYGDFRTDC	kappa	IGKV1-39*01	IGKJ2*04	15	-	-	-					
Pat TB33	180	IgG2	IGHV4-39*01	IGHJ4*02		HRSGSSDFDF	lambda	IGLV2-14*01	IGLJ3*02	22	-	-	-					
Pat TB33	189	IgA1	IGHV3-23*01	IGHJ5*02	30	ICPSQNWNFDA	kappa	IGKV3-11*01	IGKJ5*01	8	+	-	-			-		
Pat TB33	204	IgA1	IGHV4-39*07	IGHJ4*02	32	VLRGADYVDY	lambda	IGLV2-8*01	IGLJ3*02	9	-	-	+					
Pat TB33	247	IgA1	IGHV3-23*05	IGHJ4*02	34	EGYCSGGHCRYFDS	kappa	IGKV3-11*01	IGKJ5*01	20	-	-	-					
Pat TB33	302	IgG2	IGHV3-30-3*01	IGHJ3*02	19	DNDVFQKGNAFDL	lambda	IGLV2-14*01	IGLJ1*01	17	-	-	-					
Pat TB33	319	IgA1	IGHV3-21*01	IGHJ4*02	8	QLQGFDY	lambda	IGLV1-44*01	IGLJ3*02	6	-	-	+					
PatTB24	001	IgA1	IGHV1-3*01	IGHJ6*02	30	ENLRGDYGMDV	lambda	IGLV2-23*02	IGLJ1*01	13	-	+	-				-	-
PatTB24	009	IgG1	IGHV4-39*01	IGHJ3*02	0	SPRGYSYGLGAFDI	kappa	IGKV3-20*01	IGKJ4*01	0	+	-	+		-		+	+
PatTB24	017	IgA2	IGHV3-23*04	IGHJ4*02	5	VVGTIDPFDF	lambda	IGLV2-14*01	IGLJ2*01	2	-	-	-				-	-
PatTB24	023	IgG1	IGHV3-7*01	IGHJ4*02	16	DPIRSDGHNYDY	kappa	IGKV1-12*01	IGKJ5*01	19	-	+	-	-	-		-	-
PatTB24	026	IgA2	IGHV3-30*07	IGHJ6*02	23	GPICTNGDCYSYYYYGLDV	lambda	IGLV2-14*01	IGLJ3*02	13	-	-	-	-			-	-
PatTB24	037	IgA1	IGHV3-53*01	IGHJ4*02	39	RNFHY	lambda	IGLV7-46*01	IGLJ2*01	13	-	-	-	-	+	+	-	-
PatTB24	039	IgA2	IGHV3-15*01	IGHJ5*01	19	GSVEGS	lambda	IGLV7-43*01	IGLJ2*01	5	-	-	-	-		-	+	-
PatTB24	053	IgA2	IGHV3-33*01	IGHJ4*02	11	GARKSSGFLHDY	kappa	IGKV1D-33*01	IGKJ3*01	1	-	+	-				-	-
PatTB24	054	IgA1	IGHV1-46*01	IGHJ4*02	29	TIFDFWSGYSFDS	lambda	IGLV9-49*01	IGLJ2*01	12	+	+	-	+		+	+	-

TB patient	Nr	Isotype Heavy Chain	VH	JH	SHM	CDR3	Isotype Light Chain	VL	JL	SHM	MTB lysate	MTB bacteria	Cross-reactive	MTB Cell Membrane	ManLAM	BCG bacteria (FACS)	Esat-6	Ag85B
PatTB24	055	IgA2	IGHV1-8*01	IGHJ5*02	21	VSWLTSSHNWFD	kappa	IGKV1-39*01	IGKJ5*01	22	-	-	-				-	-
PatTB24	066	IgA1	IGHV3-9*01	IGHJ3*02	15	DLGYYDSGSYAFDI	kappa	IGKV3-20*01	IGKJ1*01	4	-	-	-				-	-
PatTB24	067	IgA1	IGHV3-30*19	ND	17	DGGNNNYEFYYYR	lambda	IGLV3-21*02	IGLJ1*01	5	-	-	-				-	-
PatTB24	071	IgA2	IGHV3-15*01	IGHJ4*02	11	PSAGRTVTLDY	kappa	IGKV2-28*01	IGKJ2*01	3	-	-	-				-	-
PatTB24	073	IgA1	IGHV3-23*04	IGHJ4*02	26	VNWIRDRDS	kappa	IGKV1-5*03	IGKJ1*01	10	+	-	-	+	-	-	-	-
PatTB24	077	IgG1	IGHV4-34*01	IGHJ6*02	4	ARVTLYPGVRGAYHYGMDV	kappa	IGKV1-17*01	IGKJ1*01	3	-	-	+				+	+
PatTB24	102	IgA2	IGHV4-4*07	IGHJ4*02	12	NWNWGFDS	lambda	IGLV2-8*01	IGLJ1*01	8	-	-	-				-	-
PatTB24	107	IgG4	IGHV3-33*01	IGHJ6*02	8	DGIYDFWSGYYPNNGMDV	lambda	IGLV1-40*01	IGLJ2*01	3	-	+	-				-	-
PatTB24	115	IgA2	IGHV3-23*04	IGHJ3*01	24	REITVTFWTFDV	lambda	IGLV2-14*01	IGLJ1*01	6	-	-	-				-	-
PatTB24	133	IgA1	IGHV3-11*01	IGHJ4*02	21	DIKSPGDFLYFDY	lambda	IGLV1-51*02	IGLJ3*02	4	-	-	-	-			-	-
PatTB24	159	IgG2	IGHV1-2*02	IGHJ4*02	29	DTYGGDSFDY	lambda	IGLV2-11*01	IGLJ1*01	13	-	-	-	-			-	-
PatTB24	170	IgA1	IGHV3-72*01	IGHJ5*02	24	SDHYDTNGP	kappa	IGKV1-16*02	IGKJ4*01	7	+	-	-	-		-	-	-
PatTB24	176	IgA2	IGHV4-39*01	IGHJ4*02	21	SRARAPCDY	kappa	IGKV3-20*01	IGKJ2*01	14	+	-	-	+		-	-	-
PatTB24	185	IgA2	IGHV4-39*01	IGHJ6*02	13	HPWNLFDFTGYYSGMDV	lambda	IGLV1-47*01	IGLJ2*01	7	-	+	-		-	+	-	-
PatTB24	196	IgG1	IGHV3-30*10	IGHJ4*02	32	EEWVVPNYFDY	kappa	IGKV1-39*01	IGKJ2*02	19	+	-	-	+	-	-	-	-
PatTB24	204	IgA2	IGHV3-64*05	IGHJ4*02	19	DGGAYNFPY	lambda	IGLV7-43*01	IGLJ3*02	11	-	+	-	-		-	-	-
PatTB24	210	IgA2	IGHV3-53*01	IGHJ4*02	32	VKSLAGPYFES	kappa	IGKV3-11*01	IGKJ1*01	11	+	+	-	+	-	-	-	-
PatTB24	213	IgA1	IGHV3-72*01	IGHJ3*02	20	GYSGVSIYAFDI	kappa	IGKV3-20*01	IGKJ1*01	12	-	-	-	-			-	-
PatTB24	215	IgA2	IGHV3-64*05	IGHJ4*02	22	WYGDYTY	kappa	IGKV3-20*01	IGKJ4*01	8	-	-	-	-			-	-
PatTB24	218	IgA1	IGHV1-3*01	IGHJ4*02	19	GGNYYHSSGYCLDY	lambda	IGLV3-25*02	IGLJ2*01	10	+	+	-	+	-	-	-	-
PatTB24	223	IgA1	IGHV3-43*01	IGHJ5*02	30	EAEQWLFNP	kappa	IGKV3-15*01	IGKJ1*01	5	+	-	+	+		+	+	+
PatTB24	236	IgA2	IGHV1-58*01	IGHJ6*02	14	PFSTPPPWNFGLDV	lambda	IGLV1-47*01	IGLJ3*02	10	-	-	-				-	-
PatTB24	245	IgG1	IGHV3-9*01	IGHJ4*02	6	ALAQRQYLVLVDY	lambda	IGLV1-47*01	IGLJ1*01	3	-	-	-				-	-
PatTB24	260	IgG2	IGHV4-59*08	IGHJ4*02	4	LGDYSDY	kappa	IGKV3-11*01	IGKJ2*02	1	-	-	-	-		-	-	-
PatTB24	272	IgA2	IGHV4-59*01	IGHJ4*02	25	APVDHYDSSGNHYGGDS	kappa	IGKV3-20*01	IGKJ1*01	17	-	-	-				-	-
PatTB24	275	IgG3	IGHV1-46*03	IGHJ6*02	24	PGGTYGMD	kappa	IGKV1-39*01	IGKJ2*01	15	-	-	-				-	-
PatTB24	286	IgA2	IGHV3-23*04	IGHJ4*02	17	DRGDFWRGYCFDS	kappa	IGKV1-5*03	IGKJ1*01	6	+	-	-	+	-	-	-	-
PatTB24	337	IgA1	IGHV3-23*04	IGHJ4*02	18	RGTQYGSSRPFDY	kappa	IGKV1-39*01	IGKJ4*01	9	+	-	-			-	-	-

SHM, somatic hypermutations; VH, variable segment heavy chain; JH, joining segment heavy chain; VL, variable segment light chain; JL, joining segment light chain; ND, not determined; -, non-reactive; + reactive

Appendix Table S3: Ig gene features and antibody reactivity of all expressed HBHA-reactive memory B cells from TB patients and HCWs.

Donor	Antibody Name	VH	JH	CDR3	SHM	Isotype	VL	JL	CDR3	SHM	Reactivity to HBHA	Cross-reactive	Neutralization	Enhancement
HCW001	HD001HBMem095	IGHV4-31*03	IGHJ4*02	AGSSTSYGRFDY	6	IgHM	IGKV1-12*02	IGKJ3*01	QQANSFPLFT	9	+	-	+	+
HCW001	HD001HBMem122	IGHV5-51*01	IGHJ5*02	YSGPSLYSNWFDP	12	IgHA1	IGLV1-44*01	IGLJ2*01	AAWDDSLNGPI	22	-	-		
HCW001	HD001HBMem164	IGHV4-30-2*01	IGHJ4*02	GPYGSGTVSLEY	7	IgHA1	IGLV2-8*01	IGLJ2*01	SSYAGSNNLVV	2	-	-		
HCW001	HD001HBMem216	IGHV3-23*01	IGHJ2*01	AKDPSPRRRWLQLRHLGWYFDL	19	IgHA1	IGKV2D-29*02	IGKJ1*01	MQSIEFLT	12	+	+		
HCW001	HD001HBMem238	IGHV3-74*03	IGHJ3*02	GGIGAFDF	15	IgHA1	IGLV2-8*02	IGLJ2*01	SSYAGSNNFV	7	-	-		
HCW001	HD001HBMem270	IGHV3-30*04	IGHJ4*02	GPRYSSGWYSNYFDY	20	IgHG2	IGLV2-23*02	IGLJ2*01	CSYAGNSVLL	10	-	-		
HCW001	HD001HBMem351	IGHV3-23*01	IGHJ3*02	AKPRMITMVRGDAFDI	10	IgHA1	IGKV1-27*01	IGKJ1*01	QKYNSAPHT	9	+	+		
HCW001	HD001HBMem352	IGHV3-23*04	IGHJ5*02	AKGGDSGTYYFDW	13	IgHA1	IGKV1-5*03	IGKJ1*01	QQYNSYYRT	2	-	-		
HCW001	HD001HBMem773	IGHV1-18*01	IGHJ6*03	VRAPRPDYYYFYMDV	36	IgHA1	IGLV1-47*01	IGLJ2*01	AGWDDILKCPV	23	+	+	+	
HCW001	HD001HBMem785	IGHV3-23*01	IGHJ5*01	AKMEVNGDYEYWFDS	19	IgHG2	IGKV4-1*01	IGKJ1*01	QQYYKSWT	9	-	-		
HCW001	HD001HBMem797	IGHV3-74*03	IGHJ6*03	AKPAVASAYGRPLHYYYHMDV	18	IgHA1	IGKV4-1*01	IGKJ5*01	QQCYSTPPT	10	-	-		
HCW001	HD001HBMem804	IGHV2-5*02	IGHJ5*02	GYSKQGNSYGSGSYHS	14	ND	IGKV3-11*01	IGKJ1*01	QQRSSWSE S	12	+	-	+	
HCW001	HD001HBMem823	IGHV3-74*03	IGHJ4*02	VRDRDWIVGDY	19	ND	IGLV1-47*01	IGLJ3*02	ATWDDSLGGWV	9	-	-		
HCW001	HD001HBMem825	IGHV5-10-1*03	IGHJ4*02	LLRGYYDSNAYPYYFDN	14	ND	IGKV4-1*01	IGKJ4*01	QLYYNPPLT	4	-	-		
HCW001	HD001HBMem830	IGHV4-59*01	IGHJ6*02	AKFEGLGEYYYGMDV	13	IgHA2	IGLV8-61*01	IGLJ3*02	VLYMGSGIWV	3	-	-		
HCW001	HD001HBMem835	IGHV1-2*02	IGHJ4*02	TRAHNNAYYYFDS	9	IgHA1	IGLV2-8*02	IGLJ2*01	ATWDDSLGGWV	2	-	-		
HCW001	HD001HBMem838	IGHV4-39*01	IGHJ5*02	VHFYPMVRGAMPDWFDP	17	IgHA1	IGKV3-15*01	IGKJ2*01	QQYNSWPPMYS	10	-	-		
HCW001	HD001HBMem841	IGHV5-51*01	IGHJ4*02	HLYGSGSSESDY	0	ND	IGKV1-39*01	IGKJ2*02	QQSYSTLT	2	-	-		
HCW001	HD001HBMem847	IGHV3-7*03	IGHJ4*02	DRAVSRWSEVPDY	9	ND	IGKV1-5*03	IGKJ1*01	QQYSTYSRT	4	-	-		
HCW001	HD001HBMem848	IGHV1-18*04	IGHJ4*02	DYYGSGRGDY	9	IgHA1	IGKV3-20*01	IGKJ3*01	QQYGNSPPFT	5	-	-		
HCW001	HD001HBMem854	IGHV3-21*01	IGHJ4*02	VRLVSRAAGFDY	17	IgHA1	IGKV1-27*01	IGKJ2*02	QKYNSAPYT	7	-	-		
HCW001	HD001HBMem857	IGHV3-47*01	IGHJ4*02	AKWIGGNYFGVDY	11	IGHM	IGLV7-43*01	IGLJ3*02	LLYYGGSEPWL	6	+	-	+	+
HCW001	HD001HBMem862	IGHV3-7*02	IGHJ4*02	SGTPTSGYYGGVVYAQKFDY	17	IgHA2	IGKV1-39*01	IGKJ3*01	QQIYTTRFT	17	-	-		
HCW001	HD001HBMem864	IGHV3-15*01	IGHJ6*02	TTPFRYYYGSGKNYYYGMDV	2	IgHG1	IGKV3-11*01	IGKJ4*01	QQRSNWPPLT	1	+	-		
HCW001	HD001HBMem867	IGHV3-53*01	IGHJ4*02	AHYDSSGWFDS	4	IgHA1	IGKV1-39*01	IGKJ4*01	QQSYSTLTVLT	2	-	-		
HCW001	HD001HBMem871	IGHV2-5*01	IGHJ3*02	AHSGDILTGYYNVAPFDI	4	IgHA2	IGLV1-44*01	IGLJ2*01	AAWDSSLNGPVV	6	-	-		
HCW001	HD001HBMem872	IGHV3-53*01	IGHJ4*02	AETPSGSYYGFDY	18	IgHA1	IGKV1-39*01	IGKJ4*01	QQSYR TPLT	7	-	-		
HCW001	HD001HBMem873	IGHV3-23*01	IGHJ3*01	AKDIDDSYGHRRDGWIDAFDV	16	IgHA1	IGKV3-15*01	IGKJ5*01	QQYNDWPPIT	7	-	-		
HCW001	HD001HBMem880	IGHV1-2*02	IGHJ4*02	AVDHGRNSRGFDY	13	ND	IGLV8-61*01	IGLJ3*02	VLYMGSGIWV	4	-	-		
HCW001	HD001HBMem885	IGHV4-59*01	IGHJ4*02	VIVGGTHDY	35	IgHA1	IGKV1-17*01	IGKJ3*01	LHHNSDFFT	12	-	-		
HCW001	HD001HBMem887	IGHV4-4*02	IGHJ3*02	DHSYGGDSVWAFDL	24	ND	IGKV3-20*01	IGKJ1*01	QHYHDSSWT	14	+	-	+	+
HCW001	HD001HBMem889	IGHV5-10-1*03	IGHJ2*01	QRTGGTQYWYFDL	12	IgHA1	IGLV2-23*02	IGLJ3*02	CSYAGSTTLVV	3	-	-		
HCW001	HD001HBMem899	IGHV3-23*01	IGHJ4*02	ATVLLFRGPGFLFDY	22	IgHA1	IGKV1-13*02	IGKJ4*01	QHFNGYPPT	7	-	-		
HCW001	HD001HBMem914	IGHV1-69*01	IGHJ2*01	SLPKTTVTYWYFDL	3	ND	IGKV3-20*01	IGKJ2*02	QQYGSSPQT	0	-	-		

Donor	Antibody Name	VH	JH	CDR3	SHM	Isotype	VL	JL	CDR3	SHM	Reactivity to HBHA	Cross-reactive	Neutralization	Enhancement
HCW001	HD001HBMem915	IGHV4-61*01	IGHJ4*02	GPGNYDLLTGYYPAELDY	10	IgHA1	IGLV2-8*02	IGLJ2*01	SSYAGSKNVV	3	-	-		
HCW001	HD001HBMem919	IGHV1-46*01	IGHJ6*02	SMNSGSYYFRYYYYGMDV	3	IgHA1	IGLV1-44*01	IGLJ2*01	AAWDDSLNGHVV	0	+	+		
HCW001	HD001HBMem926	IGHV4-30-4*01	IGHJ4*02	AKAVTIFPVFYFDY	0	IgHA1	IGLV1-44*01	IGLJ2*01	AAWDDSLNGHVV	1	-	-		
HCW002	T2PBMHA004	IGHV4-59*01	IGHJ1*01	DGDTSVTYFHH	19	IGHM	IGKV3-20*01	IGKJ2*03	QQYGNSPPYS	5	-	-		
HCW002	T2PBMHA007	IGHV2-5*02	IGHJ4*02	RLGGYGWNDAHFDY	12	IGHM	IGLV2-8*01	IGLJ1*01	SSNAGGSTYV	1	-	-		
HCW002	T2PBMHA014	IGHV3-48*03	IGHJ4*02	VLGLEY	9	IGHM	IGKV3-11*01	IGKJ2*02	QQRSNWPRT	14	-	-		
HCW002	T2PBMHA019	IGHV2-5*06	IGHJ4*02	TFYYDGRAEYARYSWFDP	11	ND	IGKV3-20*01	IGKJ2*01	QQYGDSPPNT	5	+	-		
HCW002	T2PBMHA036	IGHV2-5*02	IGHJ4*02	RQFLNADWNFGYFDY	13	ND	IGLV2-14*01	IGLJ3*02	SSYTGSSTV	7	+	-		
HCW002	T2PBMHA056	IGHV4-38-2*02	IGHJ6*03	DTPRAHTSTGGLVASRNWFDP	15	IGHM	IGLV1-51*01	IGLJ2*01	GTWDSSLSTGV	5	-	-		
HCW002	T2PBMHA059	IGHV4-39*07	IGHJ5*02	TRAPVVVVTVYYYYMDV	8	IGHA2	IGLV7-46*01	IGLJ1*01	LLSYSGAGV	4	-	-		
HCW002	T2PBMHA060	IGHV2-5*02	IGHJ5*02	HRGPTGVDR	21	IGHM	IGKV3-20*01	IGKJ5*01	QQYGRSPIT	19	-	-		
HCW002	T2PBMHA061	IGHV1-46*01	IGHJ4*02	RLMYSGNWDVGWFDP	28	IGHA2	IGLV7-43*01	IGLJ3*02	LLFSGGVWV	14	-	-		
HCW002	T2PBMHA065	IGHV2-5*02	IGHJ4*02	YDRSSWYFRDY	13	IGHM	IGLV2-14*01	IGLJ1*01	SSYTSSSTV	4	-	-		
HCW002	T2PBMHA072	IGHV3-7*03	IGHJ4*03	GRRDGYSYRGDDAFHI	13	ND	IGKV2-30*01	IGKJ2*01	MQLTHWPYT	6	-	-		
HCW002	T2PBMHA082	IGHV2-5*02	IGHJ4*02	DRGEWLDAYFDP	17	IGHM	IGLV2-8*01	IGLJ1*01	SSYAGSNTYV	2	-	-		
HCW002	T2PBMHA109	IGHV4-31*03	IGHJ3*01	GEGFPGMGAFDV	20	IGHA2	IGKV1-13*02	IGKJ2*01	QHFNSFPLYT	22	-	-		
HCW002	T2PBMHA116	IGHV1-46*01	IGHJ4*02	DLPGGYQGNLFDY	10	IGHA2	IGKV3-20*01	IGKJ5*01	QQYGSSPIT	6	-	-		
HCW002	T2PBMHA128	IGHV2-5*02	IGHJ4*02	DRGGDYYDSSSYFDS	13	IGHM	IGKV1-12*01	IGKJ5*01	QQAYRFPT	9	+	-		+
HCW002	T2PBMHA134	IGHV3-21*01	IGHJ6*04	PGIGVFMDV	20	IGHA1	IGKV3-20*01	IGKJ2*01	LLYINNSHRYT	11	-	-		
HCW002	T2PBMHA138	IGHV2-5*02	IGHJ4*02	PGIGVFMDV	9	IGHM	IGKV1-5*03	IGKJ1*01	QQCYSYST	11	-	-		
HCW002	T2PBMHA139	IGHV4-39*07	IGHJ4*02	RLVGGGSNWDAANFDH	17	IGHA1	IGKV1-16*01	IGKJ1*01	QQYYSYPRT	7	-	-		
HCW002	T2PBMHA142	IGHV2-5*02	IGHJ4*02	GMRYHGGD	14	IGHM	IGLV2-8*01	IGLJ1*01	SSYAGSNTYV	2	-	-		
HCW002	T2PBMHA162	IGHV2-5*02	IGHJ5*02	RLESGSPWGWWYFDH	6	ND	IGKV3-20*01	IGKJ5*01	QQYGSSPST	12	-	-		
HCW002	T2PBMHA164	IGHV1-3*01	IGHJ4*03	RLTYSGNWDVGWFDP	25	IGHM	IGKV4-1*01	IGKJ4*01	QQYYSAPLT	13	-	-		
HCW002	T2PBMHA167	IGHV5-51*01	IGHJ4*02	PRNAAIPQGWYFDC	12	IGHM	IGKV1-5*03	IGKJ2*02	QQYNTYSQT	12	-	-		
HCW002	T2PBMHA175	IGHV3-74*03	IGHJ3*02	PRNAAIPQGWYFDC	19	IGHA2	IGKV1-39*01	IGKJ3*01	QQTYGTPFT	9	+	-		
HCW002	T2PBMHA181	IGHV1-18*04	IGHJ6*02	SGGSGPILDY	34	IGHA1	IGKV3-20*01	IGKJ3*01	QQYGSSTFT	15	-	-		
HCW002	T2PBMHA184	IGHV4-34*01	IGHJ4*02	ALIDEGHGLGY	28	IGHA2	IGKV1-5*01	IGKJ2*01	HQYSDYPYT	16	-	-		
TB029	TB29PBMHA004	IGHV3-7*03	IGHJ3*02	GDYYDSSGYFHDAFDI	10	IGHM	IGKV3-15*01	IGKJ1*01	QQYNNWPPWT	11	-	-		
TB029	TB29PBMHA006	IGHV1-3*01	IGHJ6*02	AIVSTINDYYYGMDV	16	IGHM	IGKV1-27*01	IGKJ3*01	QKYNSAPL	6	-	-		
TB029	TB29PBMHA009	IGHV3-33*01	IGHJ4*02	VKPGVEYYFDY	4	IGHM	IGKV1D-33*01	IGKJ4*01	QQYDNLPLT	0	-	-		
TB029	TB29PBMHA012	IGHV3-21*01	IGHJ4*02	NFYGYYYDNSGSTKRFYFHY	20	IGHA2	IGLV1-51*01	IGLJ2*01	ATWDGSLSVL	7	-	-		
TB029	TB29PBMHA012	IGHV3-21*01	IGHJ4*02	NFYGYYYDNSGSTKRFYFHY	20	IGHA2	IGLV1-51*01	IGLJ2*01	ATWDGSLSVL	7	-	-		
TB029	TB29PBMHA015	IGHV3-33*01	IGHJ4*02	DREGGYSYGSGFFDY	1	IGHM	IGKV1-5*03	IGKJ1*01	QQYNSYPWT	3	-	-		
TB029	TB29PBMHA023	IGHV3-23*01	IGHJ4*02	DGRNYGRFDY	10	IGHA2	IGKV3-11*01	IGKJ3*01	QQWSNWPLT	6	+	+		-

Donor	Antibody Name	VH	JH	CDR3	SHM	Isotype	VL	JL	CDR3	SHM	Reactivity to HBHA	Cross-reactive	Neutralization	Enhancement
TB029	TB29PBMHA025	IGHV3-74*03	IGHJ4*02	STTGIFDY	24	IGHA2	IGKV2D-40*01	IGKJ1*01	MQRRESPST	11	-	-		
TB029	TB29PBMHA026	IGHV1-69*06	IGHJ4*02	QMGATGFDY	23	IGHA2	IGKV2-28*01	IGKJ4*01	MEALQTPH	9	-	-		
TB029	TB29PBMHA036	IGHV1-46*02	IGHJ5*02	DPRPYQRFNYFDP	36	IGHA1	IGLV1-44*01	IGLJ3*02	ASWDDSLRGLV	9	-	-		
TB029	TB29PBMHA038	IGHV3-48*03	IGHJ4*02	TGILTGLDY	28	IGHA2	IGLV1-44*01	IGLJ2*01	AAWDDSLNGRL	7	-	-		
TB029	TB29PBMHA046	IGHV1-46*01	IGHJ4*02	RSGTYWGFDY	27	IGHA1	IGKV3-20*01	IGKJ1*01	QQYASSTWT	11	+	+		
TB029	TB29PBMHA053	IGHV3-30*18	IGHJ5*02	DPRARAGLYCSSTSCYSGTQNNWFDP	0	IGHM	IGKV1-39*01	IGKJ2*01	QQSYSTPYT	0	-	-		
TB029	TB29PBMHA060	IGHV4-4*02	IGHJ3*02	DSPVRAIECSGGPCDVALDI	16	IGHA1	IGKV3-11*01	IGKJ1*01	QQRRNWPRT	7	-	-		
TB029	TB29PBMHA062	IGHV3-23*01	IGHJ4*02	ASKGCSGGSCYSVQNYFDY	7	IGHA2	IGLV2-14*01	IGLJ3*02	SSYTNSVTLV	7	-	-		
TB029	TB29PBMHA064	IGHV3-53*01	IGHJ4*02	GSPNSNEHFYLNY	32	IGHA1	IGKV1-39*01	IGKJ1*01	QQTYITPWT	20	-	-		
TB029	TB29PBMHA066	IGHV3-48*03	IGHJ4*02	EWKDDSY	17	IGHA2	IGKV3-20*01	IGKJ4*02	QHYGTSPRLT	5	-	-		
TB029	TB29PBMHA071	IGHV3-30-3*01	IGHJ6*02	DHPLYSSSSYYGMDV	21	IGHA2	IGKV1-16*01	IGKJ4*01	PWTYYGH	21	-	-		
TB029	TB29PBMHA088	IGHV4-31*03	IGHJ3*02	EVINAADSDGFDM	17	IGHA1	IGKV3-15*01	IGKJ4*01	QHYTEQPLT	5	-	-		
TB029	TB29PBMHA090	IGHV4-34*01	IGHJ6*01	AYCGGDCFMRDYYYYGVDV	42	IGHA1	IGLV3-9*01	IGLJ2*01	QVWDMSVGMI	19	-	-		
TB029	TB29PBMHA092	IGHV4-59*02	IGHJ4*02	GRDAYKTGY	25	IGHM	IGKV4-1*01	IGKJ1*01	QQYANTPWT	10	-	-		
TB029	TB29PBMHA098	IGHV4-4*02	IGHJ4*02	AYCGGDCYLDS	18	ND	IGKV2-28*01	IGKJ5*01	MQTLQTWA	6	-	-		
TB029	TB29PBMHA102	IGHV3-30*18	IGHJ3*02	GHFYDSREYSFREQRRAPDI	5	IGHA1	IGKV4-1*01	IGKJ1*01	QYHTTPET	4	-	-		
TB029	TB29PBMHA103	IGHV3-23*01	IGHJ1*01	SGCSSTSCLAEYFQH	4	IGHM	IGKV1D-33*01	IGKJ4*01	QQYDNLPT	0	-	-		
TB029	TB29PBMHA120	IGHV3-23*01	IGHJ4*02	DPNWDDGH	35	IGHA2	IGKV2-30*01	IGKJ2*02	MQASYWPLT	20	-	-		
TB029	TB29PBMHA122	IGHV3-48*03	IGHJ3*02	DRDGKSSIHAFDI	15	IGHM	IGKV1-27*01	IGKJ3*01	QKYDSAPRT	2	+	+		
TB029	TB29PBMHA135	IGHV3-7*01	IGHJ4*02	YNDGWSGHY	19	IGHA2	IGKV2-30*01	IGKJ1*01	MQASHWPPT	3	-	-		
TB029	TB29PBMHA136	IGHV1-3*01	IGHJ6*02	RQVLFYGLDV	32	IGHA2	IGLV3-25*02	IGLJ3*02	QSADNTASLWV	20	-	-		
TB029	TB29PBMHA139	IGHV1-58*01	IGHJ5*02	SGILTGSNSDS	34	IGHA2	IGKV3-20*01	IGKJ2*01	LQYHTSPYT	16	-	-		
TB029	TB29PBMHA147	IGHV2-5*02	IGHJ4*02	RVHLGFWSSAYFDY	2	IGHM	IGKV4-1*01	IGKJ1*01	QQYYTTPWT	4	-	-		
TB029	TB29PBMHA163	IGHV3-20*01	IGHJ4*02	LGPRTYDILTGPFDY	24	IGHG3	IGKV1D-33*01	IGKJ4*01	QQFDNLPLT	17	+	-		+
TB029	TB29PBMHA175	IGHV3-7*03	IGHJ4*02	DVGYFDWSDY	7	IGHM	IGLV4-69*02	IGLJ3*02	QTWGTGIRV	7	-	-		
TB029	TB29PBMHA178	IGHV3-7*01	IGHJ5*02	SAGWVSDS	23	IGHA2	IGKV3-20*01	IGKJ4*01	HQSATSPLT	17	-	-		
TB030	TB030HBMem002	IGHV3-66*01	IGHJ4*02	SGGGYYDSSGYYPFDY	7	IgHM	IGKV2-29*02	IGKJ1*01	MQGI YSWT	3	-	-		
TB030	TB030HBMem008	IGHV3-74*01	IGHJ5*02	DPHGSGSA	3	IgHM	IGLV2-14*01	IGLJ3*02	SSYTSSSTWM	3	-	-		
TB030	TB030HBMem012	IGHV3-23*01	IGHJ4*02	AKGSMAPYYFDY	21	IgHM	IGKV3-20*01	IGKJ1*01	QQYGSSRT	5	-	-		
TB030	TB030HBMem025	IGHV4-34*01	IGHJ6*02	RDPAVFRGMDV	0	IgHM	IGLV1-44*01	IGLJ2*01	AAWDDSLNGPI	28	-	-		
TB030	TB030HBMem043	IGHV3-30-3*01	IGHJ4*02	DKTTYDNSPIGY	12	IgHM	IGLV8-61*01	IGLJ3*02	VLYMGSGIWV	6	-	-		
TB030	TB030HBMem070	IGHV3-9*01	IGHJ1*01	AKDRYYSPRYFQH	0	ND	IGLV8-61*01	IGLJ3*02	VLYMGSGIWV	11	-	-		
TB030	TB030HBMem077	IGHV4-39*01	IGHJ6*02	QRHNYEVYYYGMDV	2	IgHM	IGKV1-39*01	IGKJ4*01	QQSYSTSLT	0	-	-		
TB030	TB030HBMem094	IGHV3-53*04	IGHJ6*02	YGILSYGMDV	2	IgHM	IGLV2-8*01	IGLJ2*01	SSYAGSNNFV	2	-	-		
TB030	TB030HBMem168	IGHV3-30*18	IGHJ6*02	DFFSAAGTFHYYFYGMDV	5	IgHM	IGLV1-47*02	IGLJ3*02	AAWDDSLSGVV	5	-	-		

Donor	Antibody Name	VH	JH	CDR3	SHM	Isotype	VL	JL	CDR3	SHM	Reactivity to HBHA	Cross-reactive	Neutralization	Enhancement
TB030	TB030HBMem244	IGHV4-4*02	IGHJ4*02	GAVPRKKYFDY	14	IgHA1	IGKV1-9*01	IGKJ3*01	QQVYNYPFT	7	-	-		
TB030	TB030HBMem391	IGHV3-20*01	IGHJ3*02	VRGRDYNLLHVFDI	7	ND	IGKV3-20*01	IGKJ1*01	QQSGSSPAT	4	-	-		
TB030	TB030HBMem410	IGHV3-9*01	IGHJ4*02	AKDYCSSSSCYFDY	5	ND	IGKV3-20*01	IGKJ5*01	QQYGNSPPIT	5	-	-		
TB030	TB030HBMem418	IGHV3-23*01	IGHJ4*02	VKEEPTVNAGYFDY	24	IgHA1	IGKV1D-12*01	IGKJ4*01	QQANSFPLT		-	-		
TB030	TB030HBMem421	IGHV3-74*01	IGHJ5*02	GIGDDH	8	IgHG2	IGLV4-69*01	IGLJ2*01	QTWGSGIQV	10	-	-		
TB030	TB030HBMem452	IGHV4-59*01	IGHJ4*02	GKSSSTLSYFDY	14	IgHG1	IGKV1-5*03	IGKJ1*01	QQYNDYPGT		-	-		
TB030	TB030HBMem453	IGHV5-51*01	IGHJ3*02	PMETHMLSAFDM	7	ND	IGLV8-61*01	IGLJ3*02	VLYMGSGIWV	3	-	-		
TB030	TB030HBMem462	IGHV3-30-3*01	IGHJ6*02	VVRGYSYGFAYYYGMDV	4	ND	IGLV2-14*040	IGLJ2*01	SSYTSSSTLVV	0	-	-		-
TB030	TB030HBMem466	IGHV4-39*01	IGHJ4*02	RFLYDSGSGRFDS	18	ND	IGLV8-61*01	IGLJ3*02	VLYMGSGIWV	1	-	-		
TB030	TB030HBMem470	IGHV1-18*01	IGHJ3*02	GPYYYEEAFGDAFDI	0	ND	IGKV2-30*02	IGKJ4*01	MQGTHWPPLT		-	-		
TB030	TB030HBMem471	IGHV1-2*04	IGHJ4*02	QAGSGPSFDY	14	ND	IGLV2-23*03	IGLJ2*01	CSYVGSSTSVV	4	-	-		
TB035	TB035HBMem015	IGHV3-23*01	IGHJ4*02	AKDELEYQHSYFDY	0	IgHM	IGKV1-5*03	IGKJ5*01	QQYNSYLIT	0	-	-		
TB035	TB035HBMem017	IGHV4-59*01	IGHJ6*02	SRDHWKYVKDTWAYFGLDV	35	IgHM	IGKV3-20*01	IGKJ2*01	QQYGDSQYT	27	-	-		
TB035	TB035HBMem020	IGHV2-5*02	IGHJ3*02	AHIMITFGGVMRTDAFDI	26	ND	IGLV2-11*01	IGLJ3*02	CSYAGSYSWV	8	+	-	+	-
TB035	TB035HBMem022	IGHV3-64*02	IGHJ4*02	AYYYDSRGHYLDS	11	IgHM	IGKV1-39*01	IGKJ4*02	QQTYTSS	13	-	-		
TB035	TB035HBMem042	IGHV3-74*01	IGHJ4*02	DRPHRRLDS	27	IgHG2	IGKV1-33*01	IGKJ4*01	LHHDNFPLT	12	+	-		-
TB035	TB035HBMem071	IGHV4-39*01	IGHJ6*02	AEGGYSGNYHQHYYYYGVDV	2	IgHM	IGKV3-11*01	IGKJ4*01	QQRNNWPLT	1	-	-		
TB035	TB035HBMem080	IGHV3-23*01	IGHJ6*02	VKTGGDCCWQSEYYAMDV	20	IgHA1	IGKV4-1*01	IGKJ5*01	QQYYTTPIT	11	+	-		+
TB035	TB035HBMem094	IGHV4-59*01	IGHJ5*02	DVEGDGYSQFDR	22	IgHM	IGKV3-20*01	IGKJ2*01	QQYGSSSYT	11	-	-		
TB035	TB035HBMem125	IGHV3-15*01	IGHJ4*02	NTVYTPVDQAFDY	16	IgHG2	IGKV3-15*01	IGKJ2*01	QQYNNWPPYT	6	-	-		
TB035	TB035HBMem155	IGHV3-74*03	IGHJ6*02	VRDRIEVVESVRPIYYYYGMDV	25	IgHG2	IGLV3-9*01	IGLJ2*01	QVWDSSIVI	9	-	-		
TB035	TB035HBMem159	IGHV2-5*02	IGHJ4*02	AHRAVRITWSRPYFDY	3	ND	IGKV1-33*01	IGKJ5*01	QQSNNLPPT	5	-	-		
TB035	TB035HBMem183	IGHV4-59*01	IGHJ4*02	GDVWGTD	6	ND	IGKV4-1*01	IGKJ5*01	QQYYSTPTIT	5	-	-		
TB035	TB035HBMem247	IGHV4-59*01	IGHJ6*02	GGANYYDAMDV	16	IgHG1	IGKV1-5*03	IGKJ1*01	QQYNTYRT	4	-	-		
TB035	TB035HBMem248	IGHV3-30*18	IGHJ6*02	AKEKEWLGRHYYYGMDV	20	IgHG1	IGLV2-14*03	IGLJ2*01	SSYTSSSALA	13	+	+		
TB035	TB035HBMem255	IGHV3-23*04	IGHJ6*02	GYDFLSGQPYAMEV	15	IgHA1	IGLV2-11*03	IGLJ1*01	CSYAGKFSYV	10	-	-		
TB035	TB035HBMem424	IGHV3-30*03	IGHJ4*02	PDSDWYLALFGF	21	IgHG2	IGLV2-11*03	IGLJ3*02	CSYAGAFTSL	8	-	-		
TB035	TB035HBMem447	IGHV3-7*01	IGHJ6*02	DRRQLVLGGYYYYYGMDV	4	IgHA1	IGLV1-40*01	IGLJ2*01	QSHDLGLSGSVV	3	+	+		
TB035	TB035HBMem457	IGHV3-72*01	IGHJ4*02	DFSVVGAIDYYFDS	20	IgHA2	IGLV3-1*01	IGLJ2*01	QAWERDTVT	9	-	-		
TB035	TB035HBMem465	IGHV4-59*01	IGHJ6*02	SLRFFDWLSTHPDYHYYYGMGV	4	IgHA1	IGLV2-14*04	IGLJ2*01	SSYSSSTLVV	6	-	-		
TB035	TB035HBMem467	IGHV3-9*01	IGHJ4*02	AKGPSQRYFDWLSPFDY	7	ND	IGLV2-14*01	IGLJ2*01	SSYTSSSIHVV	2	-	-		
TB035	TB035HBMem469	IGHV3-49*03	IGHJ2*01	TRKLLGHLYWYFDL	12	IgHG1	IGKV1-12*01	IGKJ1*01	QQANSFPPT	12	+	+		-
TB035	TB035HBMem470	IGHV3-74*01	IGHJ5*02	DLGIDSSAS	4	ND	IGLV3-1*01	IGLJ2*01	QAWDSRMTV	0	-	-		
TB035	TB035HBMem516	IGHV3-72*01	IGHJ4*02	VRAPYSATYFDY	16	ND	IGKV1-39*01	IGKJ4*01	QQSYSIP	10	-	-		
TB035	TB035HBMem557	IGHV1-46*01	IGHJ6*02	AIWFAFGQGSYISGSNVKDV	13	IgHG1	IGLV2-11*01	IGLJ2*01	CSYAGSYTWI	4	+	+		
TB035	TB035HBMem561	IGHV3-11*06	IGHJ5*02	VRGGVVVVADTR	19	IgHA1	IGLV3-21*03	IGLJ2*01	QVWDTNTDLGV	11	-	-		
TB035	TB035HBMem578	IGHV3-30*18	IGHJ6*02	AKVFYTRSGSPGLFYYGMDV	10	IgHG1	IGKV3-20*01	IGKJ2*01	QHYGSSPPYT	10	+	+		-
TB035	TB035HBMem584	IGHV4-39*01	IGHJ4*02	PGREDGNSIAIDY	2	IgHG2	IGLV1-40*01	IGLJ2*01	QSYDSSLSSVV	2	-	-		
TB035	TB035HBMem586	IGHV4-39*01	IGHJ5*01	SYDS	7	IgHA2	IGLV1-44*01	IGLJ1*01	AAWDDSLNGYYV	2	-	-		
TB035	TB035HBMem608	IGHV3-64*02	IGHJ4*02	DRGLHFLED	12	ND	IGLV2-14*01	IGLJ2*01	SSRTSSSTLAGV	6	-	-		

SHM, somatic hypermutations; VH, variable segment heavy chain; JH, joining segment heavy chain; VL, variable segment light chain; JL, joining segment light chain; ND, not determined; -, non-reactive; + reactive

Appendix Table S4. Functional characterization of plasmablast antibodies.

Isotype	Subclass	TB patient	Antibody Nr	VH	JH	SHM	CDR3	Isotype Light Chain	VL	JL	SHM	Mtb lysate	MTB bacteria	Cross-reactive	Cell Membrane	ManLAM	FACS BCG	Neutralization	Enhancement
IgA	IgA1	Pat TB24	037	IGHV3-53*01	IGHJ4*02	39	RNFHY	lambda	IGLV7-46*01	IGLJ2*01	13	-	-	-	-	+	+	+	-
IgA	IgA1	Pat TB24	054	IGHV1-46*01	IGHJ4*02	29	TIFDFWSGYSFDS	lambda	IGLV9-49*01	IGLJ2*01	12	+	+	-	+	ND	+	-	+
IgA	IgA1	Pat TB24	073	IGHV3-23*04	IGHJ4*02	26	VNWIRDRDS	kappa	IGKV1-5*03	IGKJ1*01	10	+	-	-	+	-	-	ND	-
IgA	IgA1	Pat TB24	170	IGHV3-72*01	IGHJ5*02	24	SDHYDTNGP	kappa	IGKV1-16*02	IGKJ4*01	7	+	-	-	-	ND	-	ND	+
IgA	IgA1	Pat TB24	218	IGHV1-3*01	IGHJ4*02	19	GGNYYHSSGYCLDY	lambda	IGLV3-25*02	IGLJ2*01	10	+	+	-	+	-	-	+	+
IgA	IgA1	Pat TB24	337	IGHV3-23*04	IGHJ4*02	18	RGTQYGSSRPFDY	kappa	IGKV1-39*01	IGKJ4*01	9	+	-	-	ND	ND	-	-	ND
IgA	IgA1	Pat TB33	189	IGHV3-23*01	IGHJ5*02	30	ICPSQNWNFDA	kappa	IGKV3-11*01	IGKJ5*01	8	+	-	-	ND	ND	-	+	ND
IgA	IgA2	Pat TB 7	066	IGHV3-15*07	IGHJ6*03	2	DNMDV	lambda	IGLV4-69*01	IGLJ3*02	8	+	-	-	-	ND	-	+	ND
IgA	IgA2	Pat TB24	039	IGHV3-15*01	IGHJ5*01	19	GSVEGS	lambda	IGLV7-43*01	IGLJ2*01	5	-	-	-	-	ND	-	+	ND
IgA	IgA2	Pat TB24	176	IGHV4-39*01	IGHJ4*02	21	SRARAPCDY	kappa	IGKV3-20*01	IGKJ2*01	14	+	-	-	+	ND	-	ND	+
IgA	IgA2	Pat TB24	185	IGHV4-39*01	IGHJ6*02	13	HPWNLFDFTGYYSGMDV	lambda	IGLV1-47*01	IGLJ2*01	7	-	+	-	ND	-	+	ND	-
IgA	IgA2	Pat TB24	210	IGHV3-53*01	IGHJ4*02	32	VKSLAGPYFES	kappa	IGKV3-11*01	IGKJ1*01	11	+	+	-	+	-	-	-	+
IgA	IgA2	Pat TB24	286	IGHV3-23*04	IGHJ4*02	17	DRGDFWRGYCFDS	kappa	IGKV1-5*03	IGKJ1*01	6	+	-	-	+	-	-	-	+
IgA	IgA2	Pat TB33	059	IGHV4-39*07	IGHJ4*02	16	YAKYSSTSYGAFDY	lambda	IGLV2-11*01	IGLJ3*02	5	+	-	-	ND	ND	-	-	-
IgA	IgA2	Pat TB33	129	IGHV4-39*07	IGHJ6*02	9	RGSGFNGMDV	kappa	IGKV3-20*01	IGKJ2*01	3	+	-	-	-	ND	-	+	+
IgA	IgA2	Pat TB33	148	IGHV3-73*02	IGHJ4*02	22	RIPNVNDF	lambda	IGLV2-14*01	IGLJ3*02	13	+	+	+	ND	ND	ND	+	ND
IgG	IgG1	Pat TB 7	017	IGHV1-69*01	IGHJ4*02	20	TAFGVVGLDFDY	lambda	IGLV7-46*01	IGLJ2*01	9	+	+	-	ND	ND	ND	ND	+
IgG	IgG1	Pat TB 7	023	IGHV1-8*01	IGHJ4*02	20	VEWYRPFDF	kappa	IGKV3-20*01	IGKJ4*01	9	+	-	+	+	-	-	ND	-
IgG	IgG1	Pat TB 7	031	IGHV3-15*07	IGHJ4*02	17	GFQGRGGWFRHITQY	kappa	IGKV1D-33*01	IGKJ3*01	8	+	+	+	ND	ND	+	ND	+
IgG	IgG1	Pat TB 7	034	IGHV4-59*08	IGHJ4*02	24	RYGGYYFDH	kappa	IGKV1D-12*01	IGKJ2*01	9	+	-	-	+	ND	-	ND	-
IgG	IgG1	Pat TB 7	067	IGHV3-21*01	IGHJ6*03	1	DNLPNRYMDV	lambda	IGLV1-51*02	IGLJ3*02	11	+	-	-	+	-	+	ND	+
IgG	IgG1	Pat TB 7	079	IGHV3-30*01	IGHJ5*02	13	PHDIVITNPFDL	kappa	IGKV1D-16*01	IGKJ4*01	9	+	+	-	+	-	-	ND	-
IgG	IgG1	Pat TB 7	196	IGHV4-34*01	IGHJ4*02	5	DVFPLRLYYYQSSGFYNY	kappa	IGKV3-20*01	IGKJ2*02	9	+	+	+	ND	-	+	ND	-
IgG	IgG1	Pat TB 7	242	IGHV3-74*01	IGHJ4*02	8	SVGSGAWRHYFDY	lambda	IGLV1-40*01	IGLJ3*02	12	+	-	-	+	ND	-	ND	+
IgG	IgG1	Pat TB 7	349	IGHV4-59*08	IGHJ4*02	21	LFYYDGSSYDPGIDY	lambda	IGLV7-43*01	IGLJ3*02	9	+	-	-	-	-	-	ND	+
IgG	IgG1	Pat TB24	023	IGHV3-7*01	IGHJ4*02	16	DPIRSDGHNYDY	kappa	IGKV1-12*01	IGKJ5*01	19	-	+	-	-	-	ND	ND	-
IgG	IgG1	Pat TB24	196	IGHV3-30*10	IGHJ4*02	32	EEWVVPNYFDY	kappa	IGKV1-39*01	IGKJ2*02	19	+	-	-	+	-	-	ND	+
IgG	IgG1	Pat TB33	052	IGHV3-21*01	IGHJ3*01	30	HILGVPGAFHV	kappa	IGKV1-NL1*01	IGKJ2*01	15	+	+	-	-	ND	-	ND	-
IgG	IgG2	Pat TB 7	317	IGHV3-33*03	IGHJ4*02	12	DYGTTGDLAVAGHLDY	lambda	IGLV1-40*01	IGLJ3*02	11	+	-	-	+	-	+	ND	-
IgG	IgG3	Pat TB 7	070	IGHV-4-34*01	IGHJ6*03	19	GSVTTDSYYYYIDV	kappa	IGKV1-6*01	IGKJ1*01	9	+	-	-	+	ND	-	ND	-
IgG	IgG3	Pat TB 7	153	IGHV3-66*02	IGHJ6*02	56	SSLCGGDCYSPVYFHYLDI	lambda	IGLV2-23*02	IGLJ1*01	11	+	+	+	ND	ND	+	ND	-

ND, not determined; SHM, somatic hypermutations; VH, variable segment heavy chain; JH, joining segment heavy chain; VL, variable segment light chain; JL, joining segment light chain.

Appendix Table S5: Statistical analysis

	exact p-value	category
**Figure 1 A,** Mann-Whitney, unpaired, non normal distribution		
MTB lysate, TB versus HD	p < 0.0001	****
Cell membrane, TB versus HD	p < 0.0001	****
CFP, TB versus HD	p=0.0066	**
**Figure 1 B,** Mann-Whitney, unpaired, non normal distribution		
MTB lysate, TB versus HD	p=0.0006	***
Cell membrane, TB versus HD	p=0.0020	**
CFP; TB versus HD	p=0.1186	ns
**Figure 2 E,** Mann-Whitney, unpaired, non normal distribution	p < 0.0001	****
**Figure 5 B,** Mann-Whitney, unpaired, non normal distribution		
IgA vs IgG	p<0.0001	****
**Figure 5 D,** Mann-Whitney, unpaired, non normal distribution		
IgA vs IgG	P=0.0061	**
**Figure 5 E,** t-test, non-paired, normal distribution as tested by D'Agostino-Pearson omnibus test		
TB24PB054, IgA vs IgG	P=0.0063	**
TB24PB037, IgA vs IgG	P=0.0079	**
TB33PB129, IgA vs IgG	P=0.0014	**
TB24PB218, IgA vs IgG	P=0.0170	*
HCW1HBMem095, IgA vs IgG	P=0.0017	**
**Figure 6 A,** Mann-Whitney, unpaired, non normal distribution		
no mAb vs IgA TB24PB037	P=0.0005	***
no mAb vs IgG TB24PB037	P= 0.9119	ns
**Figure 7 B,** Mann-Whitney, unpaired, non normal distribution		
IgA vs IgG	p<0.0001	****

Appendix Figure S1 A and B

A

B

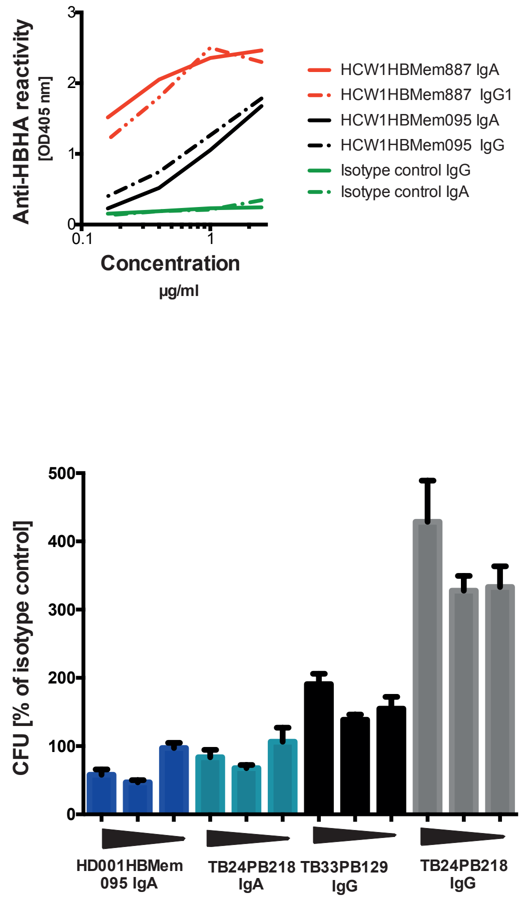

Appendix Figure S1. Antigen-reactivity and concentration-dependent effect of monoclonal IgG and IgA antibodies on MTB infection of human lung epithelial cells.
(**A**) Representative HBHA ELISA data from two independent experiments for the indicated IgA and IgG antibodies and respective isotype controls. (**B**) Relative bacterial counts (CFU) compared to the medium control (100%) in human lung epithelial A549 cells 1 h after infection with MTB, pre-incubated with the indicated recombinant MTB-reactive antibodies at different concentration of 100 µg/ml, 10 µg/ml and 1 µg/ml (from left to right). Pooled data from one or two independent experiments with two or three technical replicates each. Mean and SD are indicated.
