# Supplementary material for: Human isotype‐dependent inhibitory antibody responses against Mycobacterium tuberculosis
Source: EMBO Mol Med. 2016 Oct 11;8(11):1325–39. doi: 10.15252/emmm.201606330 (PMC5090662; doi:10.15252/emmm.201606330)
Supplement: Supplementary file 2 — Expanded View Figures PDF [file EMMM-8-1325-s002.pdf]

Expanded View Figures

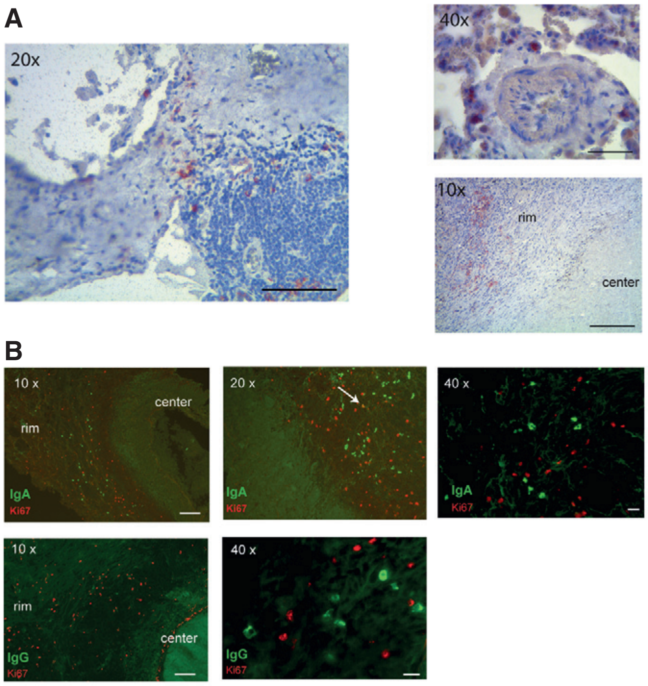

Figure EV1. IgA$^+$ and IgG$^+$ plasmablasts/cells in the lung of TB patients.

A Immunohistochemistry staining for CD138 of infected lung sections from TB patients. Images were taken at different magnifications as indicated. The rim and the center of one granuloma are indicated in the lower right picture.

B (Top) Immunofluorescence staining for IgA (green) and Ki67 (red) of infected lung sections of one representative out of three analyzed TB patients. White arrow indicates one Ki67/IgA double-positive cell. (Bottom) Immunofluorescence staining for IgG (green) and Ki67 (red).

Data information: Different magnifications as indicated. Scale bars, 10 μm for 40× magnification or 100 μm for 10× and 20× magnifications. Data are representative of two independent experiments with samples from three different TB patients.

 © 2016 The Authors
