# Supplementary figures and images for: Human isotype‐dependent inhibitory antibody responses against Mycobacterium tuberculosis
Source: EMBO Mol Med. 2016 Oct 11;8(11):1325–39. doi: 10.15252/emmm.201606330 (PMC5090662; doi:10.15252/emmm.201606330)

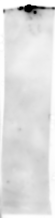

Supplement: Supplementary file 4 — Source Data for Figure 4 [file EMMM-8-1325-s003.zip › Source_Data_Fig4/negative_control.tiff]

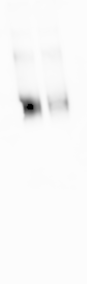

Supplement: Supplementary file 4 — Source Data for Figure 4 [file EMMM-8-1325-s003.zip › Source_Data_Fig4/positive_control.tiff]

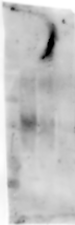

Supplement: Supplementary file 4 — Source Data for Figure 4 [file EMMM-8-1325-s003.zip › Source_Data_Fig4/TB24PB037.tiff]

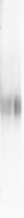

Supplement: Supplementary file 4 — Source Data for Figure 4 [file EMMM-8-1325-s003.zip › Source_Data_Fig4/TB33PB123_ab.tiff]
